# Supplementary material for: MicroRNAs as a Suitable Biomarker to Detect the Effects of Long-Term Exposures to Nanomaterials. Studies on TiO2NP and MWCNT
Source: Nanomaterials (Basel). 2021 Dec 20;11(12):3458. doi: 10.3390/nano11123458 (PMC8707110; doi:10.3390/nano11123458)
Supplement: Supplementary file 1 [file nanomaterials-11-03458-s001.zip › nanomaterials-1452912-supplementary.pdf]

supplementary materials

MicroRNAs as a Suitable Biomarker to Detect the Effects of Long-Term Exposures to Nanomaterials. Studies on TiO_2NP and MWCNT

Sandra Ballesteros [1], Gerard Vales [2], Antonia Velázquez [1], Susana Pastor [1], Mohamed Alaraby [1,3], Ricard Marcos [1,*] and Alba Hernández [1,*]

[1] Group of Mutagenesis, Department of Genetics and Microbiology, Faculty of Biosciences, Universitat Autònoma de Barcelona, 08193 Cerdanyola del Vallès, Spain; sandra.ballesteros@uab.es (S.B.); antonia.velazquez@uab.es (A.V.); susana.pastor@uab.es (S.P.); ma_abdalaziz24@yahoo.com (M.A.)

[2] Finnish Institute of Occupational Health, 00250 Helsinki, Finland; gerard.vales@ttl.fi

[3] Zoology Department, Faculty of Sciences, Sohag University, Sohag 82524, Egypt; mohamed.alaraby@science.sohag.edu.eg (M.A.)

* Correspondence: ricard.marcos@uab.cat (R.M.); alba.hernandez@uab.cat (A.H.)

Supplementary figures

Figure S1. MicroRNA expression changes of BEAS-2B cells exposed to the lowest concentration of TiO_2NPs. (A) Deregulated microRNAs at week 3 (1 + 2) and week 6 (4 + 2) after the exposure to TiO_2NPs. Data are plotted as mean, and error bars represent the SEM. (B) Venn diagram showing the number of microRNAs significantly deregulated at week 3 (1 + 2) and week 6 (4 + 2) of the exposure. The overlapping area indicates the number of microRNAs commonly deregulated at both exposure times. Overexpressed microRNAs are in bold and underlined. Results were analyzed with the Student's *t*-test ($P < 0.05$).

Figure S2. MicroRNA expression changes of BEAS-2B cells exposed to the lowest concentration of MWCNTs. (A) Deregulated microRNAs at week 3 (1 + 2) and week 6 (4 + 2) after the exposure to MWCNTs. Data are plotted as mean, and error bars represent the SEM. (B) Venn diagram showing the number of microRNAs significantly deregulated at week 3 (1 + 2) and week 6 (4 + 2) of the exposure. The overlapping area indicates the number of microRNAs commonly deregulated at both exposure times. Overexpressed microRNAs are in bold and underlined. Results were analyzed with the Student's *t*-test ($P < 0.05$).

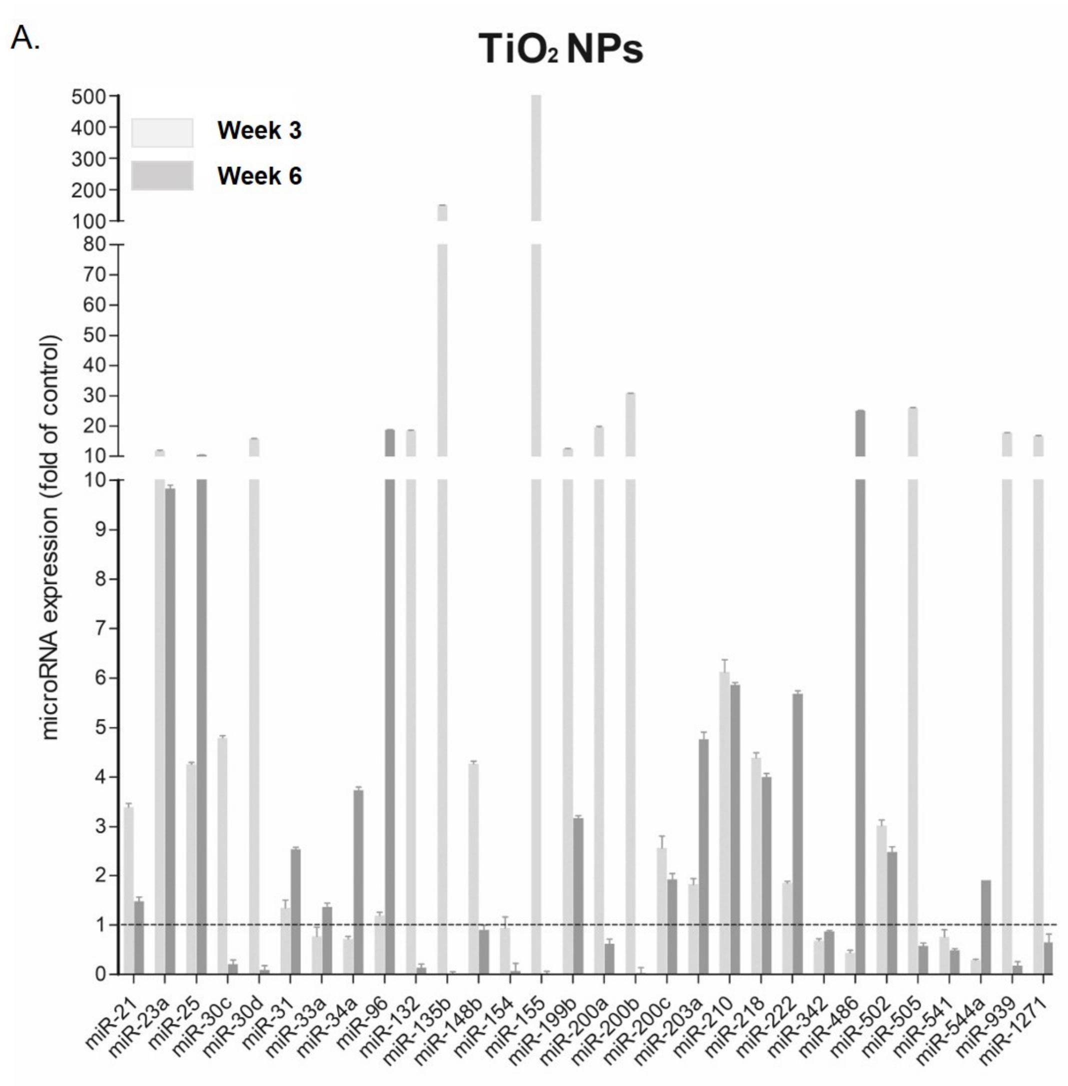

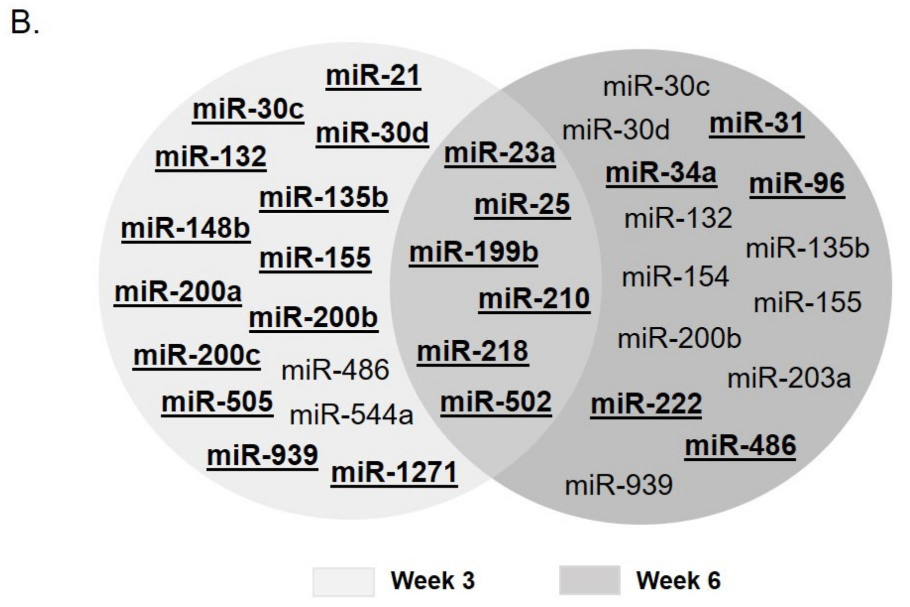

Figure S1: MicroRNA expression changes of BEAS-2B cells exposed to the lowest concentration of TiO_2NPs.

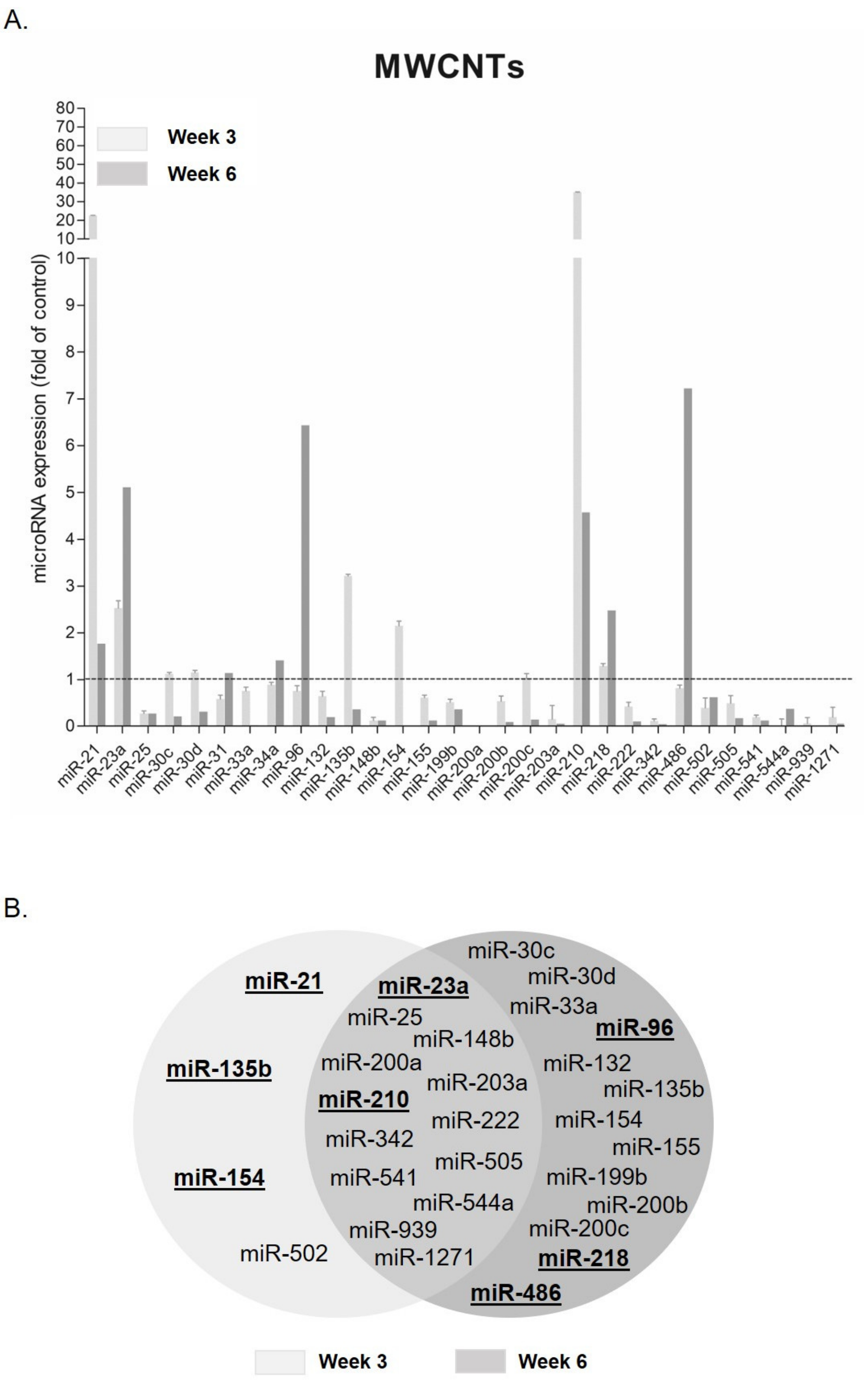

Figure S2: MicroRNA expression changes of BEAS-2B cells exposed to the lowest concentration of MWCNTs.
